# Speculation of the Time-Dependent Change of FIB4 Index in Patients with Nonalcoholic Fatty Liver Disease: A Retrospective Study

**DOI:** 10.1155/2018/5323061

**Published:** 2018-03-12

**Authors:** Hiroshi Miyata, Satoru Miyata

**Affiliations:** Sugimoto Clinic, 3-2-27 Yamanouchi, Sumiyoshi-ku, Osaka city, Osaka-fu 558-0023, Japan

## Abstract

**Aim:**

To speculate on the time-dependent change of FIB4 index in patients with nonalcoholic fatty liver disease (NAFLD) and its increase-decrease rate per year, simply and accurately.

**Methods:**

In all 23 patients with NAFLD with the value of FIB4 index over 1.30 at the peak, the period from the first to each examination date was calculated and this period (years) was regarded as *x*. Next, the mean value of FIB4 index during the past year to each examination date was regarded as *y*. In every *y*, the minimum and the maximum *y* value were found out. Between *x* corresponding to this minimum *y* and *x* corresponding to this maximum *y*, the correlation between *x* and *y* was analyzed as the main subject. Then, the slope of each correlation was investigated, because it should indicate increase-decrease rate per year.

**Results:**

In all 23 patients, the correlations as the main subject were recognized and the mean absolute value of correlation coefficient (*r*) was 0.91 ± 0.08. As for the slope, the mean absolute value was 0.1371 ± 0.1147 (/year).

**Conclusion:**

Simply and accurately, the time-dependent change of FIB4 index and its increase-decrease rate per year could be approximately speculated.

## 1. Introduction

Nonalcoholic fatty liver disease (NAFLD) is one of the most common causes of chronic liver disease worldwide [1–5]. A liver biopsy still remains the gold standard for the diagnosis of nonalcoholic steatohepatitis (NASH), but it is difficult to perform liver biopsies in all patients with NAFLD. Therefore many noninvasive methods for estimating liver fibrosis have been developed; these are direct markers and the scoring systems, such as type IV collagen 7S [6, 7], hyaluronic acid [8, 9], aspartate aminotransferase (AST)/alanine aminotransferase (ALT) ratio (AAR) [9, 10], NAFLD fibrosis score [11], BARD score [12], NAFIC score [7], and so on.

FIB4 index has been developed to predict liver fibrosis in patients with HIV/HCV coinfection [13] and it is also useful for estimating liver fibrosis in patients with NAFLD [14–16]. However there were few reports analyzing the transition of FIB4 index during all the clinical period in patients with NAFLD. Probably for the dispersion of the data, it has been difficult to estimate the accurate value.

In this study the correlation between the period from the first to each examination date and the mean value of FIB4 index during the past year to each examination date was analyzed. This correlation was thought to be the time-dependent change of the mean FIB4 index during the past one year and in the present study the correlation was proved to be extremely strong. Moreover, increase-decrease rate per year could be derived from the slope of the correlation in the scatter diagram.

In this retrospective study, the aim was to speculate approximately on the time-dependent change of FIB4 index and its increase-decrease rate per year, simply and accurately.

## 2. Methods

### 2.1. Patients

A total of 23 patients between October 1999 and June 2017 were enrolled with the following criteria: negative HBs antigen, negative HCV antibody, and negative anti-mitochondrial antibody [17]. Serum CRP levels were continuously negative. Patients whose values of anti-nuclear antibody (ANA) showed more than 1 : 160 were excluded [18]. The peak ALT levels were over 40 (U/L) for males or over 30 (U/L) for females [19–21]. The peak value of FIB4 index was over 1.30 [14, 15, 22] in every patient. Fatty liver was diagnosed with ultrasonography and/or computed tomography. Drug induced liver injury and hereditary liver diseases were denied by the interview. Patients who consumed alcohol over 30 g per day for males or over 20 g per day for females were excluded [14, 23, 24]. Patients whose observed period in the clinic was less than two years were excluded. Finally patients whose maximum interval between examinations was more than one year were excluded.

All procedures in this study were conducted with the declaration of Helsinki (1964). The written informed consent was not applicable, because this is a retrospective study. In this study, direct data of AST, ALT, age, and platelet count were only used in patient characteristics and it was not possible to identify individuals.

### 2.2. Correlations

#### 2.2.1. The Main Correlations

First, the period from the first to each examination date was calculated and this period (years) and was regarded as *x*. Next, the mean value of FIB4 index during the past one year to each examination date (the mean FIB4 index YTD) was regarded as *y*. Because of using the mean value during the past one year as *y*, *x* less than 1.00 (years) and *y* corresponding to this *x* were excluded; the minimum *x* value in every *x* was more than 1.00 (years). In every *y*, the minimum *y* value and the maximum *y* value were found out. Between *x* corresponding to this minimum *y* and *x* corresponding to this maximum *y*, the correlation between *x* and *y* was analyzed in every patient. This correlation was defined as the main correlation. There are two possibilities; either the values of correlation coefficient (*r*) are positive or these are negative. The group with positive value of *r* was defined as FIB4 index-increasing group and the group with negative value of *r* was also defined as FIB4 index-decreasing group.

#### 2.2.2. The After-Main Correlations

Then, another correlation was analyzed, except for the data during the period of the main correlation. However, both ends of the data in the main correlation were not excluded.

After the period of the main correlation, it was checked whether the period to the last examination date was more than 1.00 (years) or not. Only when this period was more than one year, the analysis was performed. The first data of this analysis was automatically the last data in the main correlation. In FIB4 index-increasing group, the minimum *y* value was newly found out in this period. Yet, in FIB4 index-decreasing group, the maximum *y* value was newly found out. In both groups, from the maximum *x* in the main correlation to *x* corresponding to *y* newly found out, the correlation between *x* and *y* was analyzed. This correlation was defined as the after-main correlation.

#### 2.2.3. The Before-Main Correlations

Finally, before the period of the main correlation, it was checked whether the period from the first examination date was more than 2.00 (years) or not, because *x* less than 1.00 (years) had been excluded. Only when this period was more than two years, the analysis was performed. The last data of this analysis was automatically the first data in the main correlation. In FIB4 index-increasing group, the maximum *y* value was newly found out in this period. Yet in FIB4 index-decreasing group, the minimum *y* value was newly found out. In both groups, from *x* corresponding to *y* newly found out to the minimum *x* in the main correlation, the correlation between *x* and *y* was analyzed. This correlation was defined as the before-main correlation.

#### 2.2.4. A Total of the Correlations Recognized in the Study

The cumulative correlations recognized in this study were shown.

### 2.3. Slopes of Correlations

In every patient, increase-decrease rate per year of the mean FIB4 index YTD was derived from the slope of the main correlation. In the same way it was also derived from each slope of the after-main correlation and/or the before-main correlation, if these correlations were recognized.

### 2.4. Statistics Analysis

Each correlation between two parameters was evaluated by Pearson's correlation. A *p* value (*p*) less than 0.05 was considered statistically significant. It was conducted by Microsoft Excel for MAC 2011.

## 3. Results

### 3.1. Patient Characteristics

12 out of 23 patients (52.2%) were male. In 20 patients the values of ANA were less than 1 : 40 [18] and in three patients these were 1 : 40, 1 : 40, and 1 : 160, respectively, and the values of anti-smooth muscle antibody were all less than 1 : 40 and also immunoglobulin G levels were all within the upper normal limit of the clinic [25]. In 17 patients computed tomography scans were performed. In all patients, the mean value of the peak ALT levels was 72 ± 35 (U/L) and that of the peak value of FIB4 index was 2.84 ± 1.34. In 10 patients the peak values of FIB4 index were more than 2.67 [14, 21], yet in nine patients those were less than 2.00. In all patients, the mean value of platelet count at the bottom was 165 ± 45 (×10^9^/L). Of 23 patients, 19 consumed no alcohol and the remaining four were all males (Table 1).

### 3.2. Correlations

#### 3.2.1. The Main Correlations

**Figure 1:**
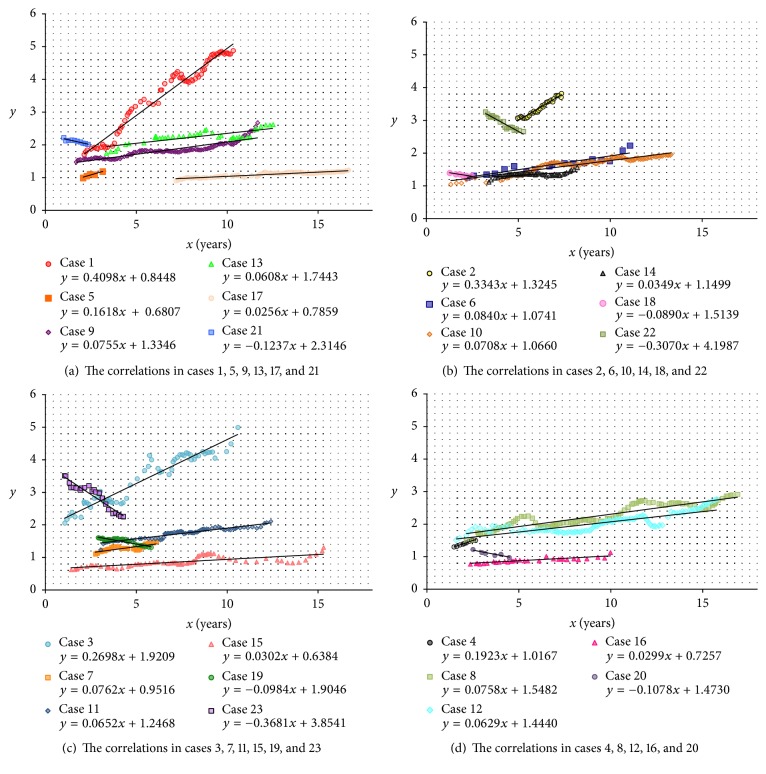
The correlation between the period from the first examination to each examination (*x*) and the mean value of FIB4 index during the past year to each date of examination (*y*) in the phase of all 23 main correlations.

In all 23 patients the main correlations were recognized (Figure 1) and the mean absolute value of *r* was 0.91 ± 0.08 (Table 2). Each *p* was shown in Table 2. Of 23 patients, 17 were categorized in FIB4 index-increasing group and the mean value of *r* was 0.90 ± 0.09 (0.69 to 0.99). In 11 of these 17, the values of *r* were more than 0.90. On the other hand, six of 23 patients were categorized in FIB4 index-decreasing group and the mean value of *r* was −0.94 ± 0.02 (−0.97 to −0.91). In all these six patients, the absolute values of *r* were more than 0.90. Therefore, in 17 out of 23 patients, the absolute values of *r* were more than 0.90. In a total of 23 patients, the mean value of interval between examinations was 0.17 ± 0.09 (years), that is, 64 ± 33 (days), and the mean value of the total clinical period was 10.7 ± 4.6 (years) (Table 2). Since *x* less than 1.00 (years) were excluded, the total analyzed period was 9.5 ± 4.5 (years) (Table 2). The period in which the main correlation was recognized (the main correlation's period) was 6.6 ± 4.5 (years) and the mean ratio of the main correlation's period to the total analyzed period was 64 ± 23% (27% to 98%).

#### 3.2.2. The After-Main Correlations

In 11 out of all 23 patients, each period to the last examination after the main correlation was more than 1.00 (years). In eight of these 11, the after-main correlations were seen. The mean absolute value of *r* was 0.93 ± 0.04 and each *p* was shown in Table 3. In the remaining three of these 11, that is, in patients of cases 5, 16, and 18, the correlations were not recognized statistically. In these three patients, numbers of analyzed data were four, five, and five, respectively, and the correlations were not recognized by *p* = 0.17 and *r* = −0.83, by *p* = 0.09 and *r* = −0.82, and by *p* = 0.07 and *r* = 0.85, respectively (Table 3).

#### 3.2.3. The Before-Main Correlations

In 14 out of all 23 patients, each period from the first examination before the main correlation was more than 2.00 (years). In 10 of these 14, the before-main correlations were seen. The mean absolute value of *r* was 0.95 ± 0.05 and each *p* was shown in Table 3. In the remaining four of these 14, that is, in patients of cases 6, 8, 11, and 20, the correlations were not recognized statistically. In two patients of cases 6 and 11, numbers of analyzed data for the correlations were both two and it was impossible to analyze. In the remaining two patients of cases 8 and 20, numbers of analyzed data were three and four and the correlations were not recognized by *p* = 0.34 and *r* = −0.86 and by *p* = 0.27 and *r* = 0.73, respectively (Table 3).

#### 3.2.4. A Total of the Correlations Recognized in the Study

The cumulative number of all correlations recognized in this study was 41 (Table 3). The mean absolute value of *r* was 0.92 ± 0.07. In 32 of 41 correlations the absolute values of *r* were over 0.90 and in only three of 41 they were less than 0.80 (0.688 to 0.799).

### 3.3. Slopes of Correlations

**Figure 2:**
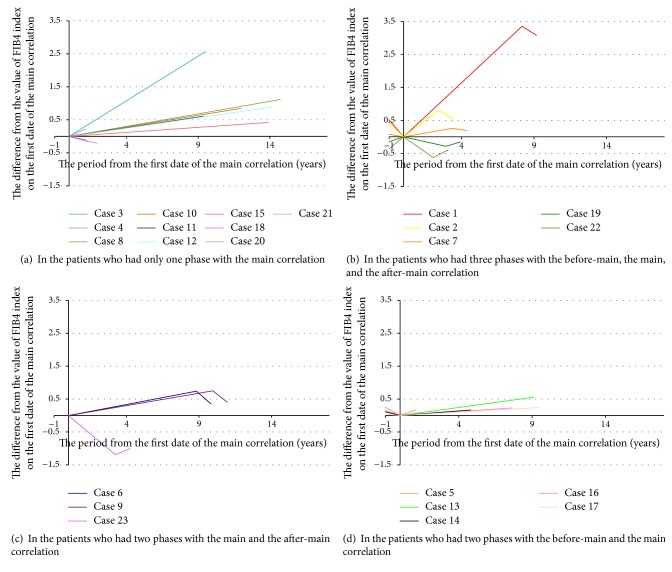
The slopes of all 41 correlations recognized in this study. All the main correlations were shown without *y*-intercept. (b, c, d): the before-main and the after-main correlations were shown only for a year.

In all 23 main correlations, the values of increase-decrease rate per year of the mean FIB4 index YTD were shown as the slope in Table 2. In them the mean absolute value of the slope was 0.1371 ± 0.1147 (/year). In 17 correlations categorized in FIB4 index-increasing group, the mean value of the slope was 0.1212 ± 0.1114 (/year), yet in six ones categorized in FIB4 index-decreasing group, it was −0.1823 ± 0.1117 (/year). Then, in a total of 41 correlations, the mean absolute value of the slope was 0.1764 ± 0.1307 (/year). In 22 positive correlations, the mean value of the slope was 0.1415 ± 0.1118 (/year), yet in 19 negative correlations, it was −0.2168 ± 0.1319 (/year). All 41 correlations were shown in Figure 2. In order to demonstrate the slopes clearly, the main correlations were shown without *y*-intercept in Figure 2.

## 4. Discussion

**Figure 3:**
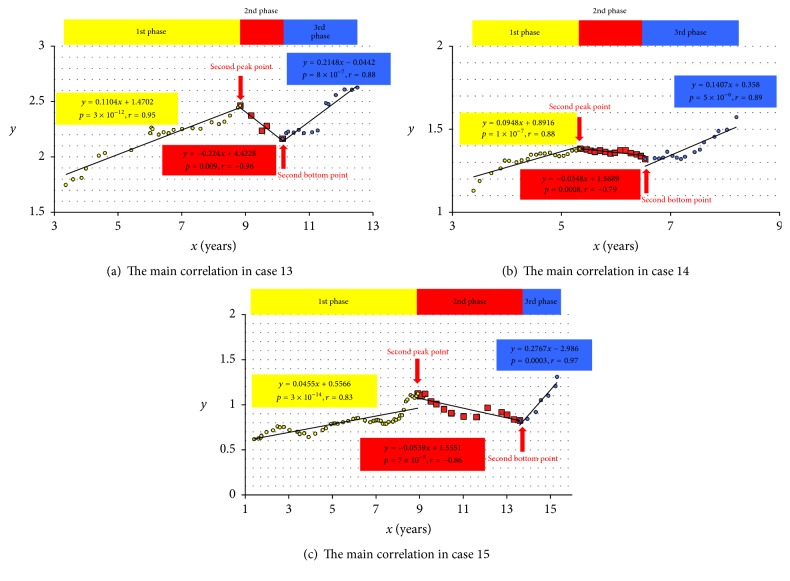
All the three correlations with the absolute value of *r* less than 0.80. The value of *x* was defined as the period from the first to each examination date and also the value of *y* was defined as the mean value of FIB4 index during the past one year to each examination date. In all the three correlations, values of *y* gradually increased and then at once reached the peak, which was so-called “second peak point.” After this point to the last point of data in each correlation, there was the bottom point, which was so-called “second bottom point.”

In the present study the correlations between the period from the first to each examination and the mean FIB4 index YTD were analyzed. The results just would mean the time-dependent change of the mean FIB4 index YTD. All 23 enrolled patients had at least one phase with the main correlation (Figure 1 and Table 2) and the mean absolute value of *r* was 0.91 ± 0.08. In 17 of these 23 (74%) the absolute values of *r* were over 0.90. Meanwhile, 10 of 23 patients had only one phase with the main correlation (Figure 2(a)) and the remaining 13 had several phases (Figures 2(b)–2(d)). As a result, a total of 41 correlations were recognized in the study and the mean absolute value of *r* was 0.92 ± 0.07. In 32 of all the 41 correlations (78%) the absolute values of *r* were over 0.90 (Table 3). In addition, the authors will show the reason why there were some correlations with low absolute values of *r*. For example, out of all 41, three correlations (7.3%) with the absolute values of *r* less than 0.80 were recognized and all of them were the main correlations. They were enumerated in Figure 3. In all the three correlations the mean FIB4 index YTD gradually increased and then at once reached the peak, which was so-called “second peak point.” After this point to the last point of data in the correlation, there was the bottom point, which was so-called “second bottom point.” In these correlations the mean FIB4 index YTD moved like an italic type of “*N*.” In such a condition, the absolute value of *r* would probably become low.

In statistics, the coefficient of determination, which is calculated as a squared value of *r*  (*r*^2^), determines how enough the outcomes could be explained by the hypotheses. In this study, the mean value of *r*^2^ in the main correlation was 0.83 ± 0.13 and that in a total of 41 correlations was 0.86 ± 0.12. The value of *r*^2^ like these could not be ignored, even if the number was 23 or 41. Moreover, it had been explained why there were some correlations with low absolute values of *r*. Statistically it was thought to be sufficient to speculate how the mean FIB4 index YTD moved.

There was another important thing about the movement of the mean FIB4 index YTD. In three of five patients with three phases (Figure 2(b)), the mean FIB4 index YTD showed decreasing firstly, increasing secondly, and decreasing finally, yet in two of these five (Figure 2(b)), it showed increasing, decreasing, and increasing. On the other hand, in eight patients with two phases (Figures 2(c)-2(d)), it showed firstly decreasing and finally increasing or firstly increasing and finally decreasing. From the viewpoint of the movement, the most important thing was that there was a turning point in which the mean FIB4 index YTD changed from increasing to decreasing or from decreasing to increasing. This means that the mean FIB4 index YTD moved like a wave. Even in the main correlations, these waves were seen and the typical ones had been picked up in Figure 3.

Now, developing this study's methods, there would be a possibility. The possibility is that the methods will be applicable for any partial period. In order to validate it, the analysis only has to be performed, not from the first examination date and/or not to the last examination date. For example, in all the 23 patients the period was newly set from the closest date after half the total clinical period to that date after three-quarters. Limiting to this period, the analysis was newly performed through this study's methods. Out of 23 patients, seven whose analyzed period remained less than two years were excluded, because it was necessary for a year to calculate the mean FIB4 index YTD. In all the remaining 16 out of 23, the new main correlations were analyzed. In the patient of case 16 there were only two pieces of data and it was impossible to analyze the correlation and in the patient of case 13 there were three pieces of data and the correlation was not statistically recognized by *p* = 0.08 and *r* = 0.99. However, in the remaining 14 patients the new main correlations were recognized and the mean absolute value of *r* was 0.92 ± 0.05. In addition, in all 14, the absolute values of *r* were over 0.80. From this result, it was thought that the methods might be applicable for any partial period (Table 4).

**Figure 4:**
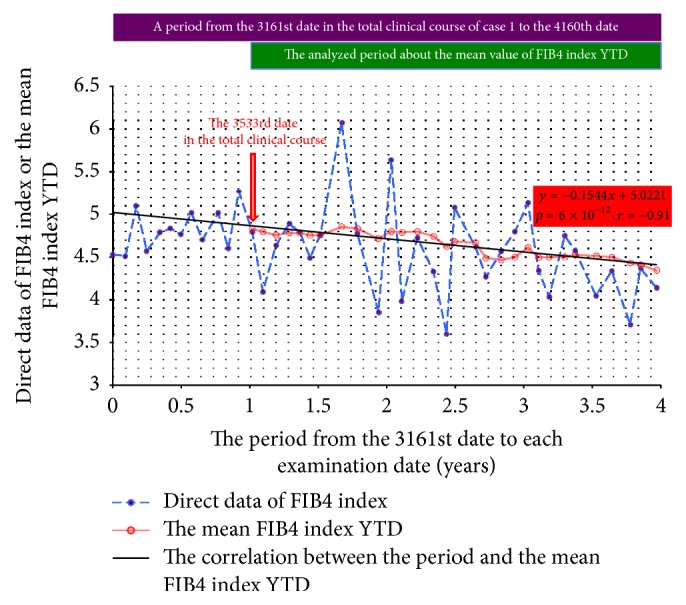
The transition of FIB4 index in a partial period in case 1. In a patient of case 1, a correlation was analyzed in a partial period newly set. The period was set from the 3161st date in the total clinical course to the 4610th date. After the earliest date more than a year from the 3161st date, that is, the 3533rd date finally to the 4160th date, the correlation between the period (years) from the 3161st date and the mean FIB4 index YTD was analyzed through this study's method. All the data were cited from our previous case report [26]. The mean FIB4 index YTD, the mean value of FIB4 index during the past one year.

The trigger of the start of the present study was a case report of a patient with NAFLD that the authors have already reported previously [26]. In this report we analyzed the correlation in a partial period between time (years) and the direct data of FIB4 index and we showed in that period that the direct data of FIB4 index decreased with the rate of 0.15 per year, statistically proven by the general linear regression model; FIB4 index = 4.90 – 0.15 × time (years) (*p* = 0.02). This patient was enrolled as a patient of case 1 in the present study. If this study's methods were applicable for any partial period, similar outcome should be obtained. As expected, the mean FIB4 index YTD decreased with the rate of 0.15 per year (Figure 4); the mean FIB4 index YTD = 5.02 − 0.15 × time (years) (*p* = 6 × 10^−12^ and *r* = −0.91). This was very similar to our previous formula and *p* has become extremely low.

In this way, it was very easy to estimate increase-decrease rate per year of the mean FIB4 index YTD. It was the value of the slope of each correlation on the scatter diagram. As for the main correlations, the mean absolute rate per year was 0.1371 ± 0.1147 (Table 2). In this viewpoint, since the difference of FIB4 index between 1.30 and 2.67 [14, 27] is 1.37, it would take about 10 years by the mean absolute rate per year.

Meanwhile, the limitations of this study should be shown. Firstly, interval between examinations in this study was 0.17 ± 0.09 (years) and it would be rather short. If the interval was longer, the dispersion of the data could not be minimized and the strong correlation might not be recognized. In fact, the after-main correlations or the before-main correlations were not recognized statistically in some patients, probably for the lack of number of data. About this, if the interval in the correlations had been shorter, the number of data would have increased and the correlation might have been seen. Anyway, when there were at least six pieces of data for the analysis, the correlations were all recognized in this study.

Secondly, it took a year from the first examination to calculate the data and also took another year to analyze the data.

Thirdly, in this study histological findings were not performed. Certainly, it was speculated that, compared to the earlier studies, liver fibrosis in the patients of this study would be rather advanced. Several proofs should be shown as follows. In this study the mean value of FIB4 index at the peak was 2.84 ± 1.34 (Table 1). Shah et al. reported that, for advanced fibrosis (stage 3-4), a FIB4 > or = 2.67 had an 80% positive predictive value [14]. Moreover, the mean value of platelet count at the bottom was 165 ± 45 (×10^9^/L) (Table 1). Kaneda et al. reported that the platelet count was found to be an independent predictor of cirrhosis and a cut-off value of 16 × 10^4^/microL for the platelet count was associated with an optimal combination of sensitivity (100%) and specificity (95%) [8]. In addition, the mean value of type IV collagen 7S at the peak was 5.2 ± 2.0 (ng/mL) (Table 1). It was reported that in patients with NASH the type IV collagen 7S domain was significantly elevated in patients with advanced fibrosis by multiple regression analysis [6] and Sumida et al. have developed NAFIC score in biopsy-proven patients with NAFLD to differentiate NASH from NAFLD, using the cut-off of type IV collagen 7S ≥ 5.0 (ng/mL) [7]. Then, the mean value of M2BPGi at the peak was 1.09 ± 0.86 (Table 1). Nishikawa et al. reported that in NASH patients the median values in each fibrosis stage were 0.7 COI in F1, 0.7 COI in F2, 1.2 COI in F3, and 2.4 COI in F4 [28] and Lai et al. also reported that the AUROC of the COI for the diagnosis of fibrosis stages ≥1, ≥2, ≥3, and 4 was 0.61, 0.71, 0.74, and 0.84, respectively [29]. These facts would show liver fibrosis in the enrolled patients would be rather advanced.

However, it is a problem whether the time-dependent change of FIB4 index corresponds to that of fibrosis by a liver biopsy. About this there was a retrospective study. McPherson et al. reported the following [30]. In 108 patients who had serial biopsies (median interval 6.6 years, range 1.3–22.6), there was a significant relationship between the change in fibrosis between biopsies and the change in both NAFLD fibrosis score [11] and FIB-4 score. They compared patients with histological evidence of increasing fibrosis stage (progressors) to subjects whose fibrosis remained stable or regressed (nonprogressors) and in progressors FIB-4 score was changed from 1.85 ± 1.31 at baseline biopsy to 2.33 ± 1.69 at follow-up one, yet in nonprogressors it was changed from 1.26 ± 0.57 to 1.36 ± 0.62 [30].

Nevertheless, there were few studies about the relationship between change in FIB4 index and change in fibrosis conducted by paired biopsies and therefore further verifications should be done. However, in the process of verifying it, this study's methods might be useful, because the dispersion of a single direct data of FIB4 index probably would be a considerable problem. It is difficult to perform biopsies to all patients with NAFLD and in such a condition the methods to minimize the dispersion of the data would be helpful.

To consider the risk of liver fibrosis based on grasping the whole picture of the movement of the mean FIB4 index YTD would be one of the practical benefits in the study. For all practical purposes, the latest correlation means either the main correlation or the after-main correlation and if the value of the slope of that correlation were positive, the progression of liver fibrosis would be concerned. Especially in a patient whose last data of the mean FIB4 index YTD in that correlation shows a value over 2.67, advanced liver fibrosis should be well considered. On the other hand, in a patient with a negative value of the slope of the latest correlation, even if the last data of the mean FIB4 index YTD shows a value over 2.67, it would be a little difficult to assess the risk. In fact, in a patient of case 1, a sever complication was gradually improved in such a condition [26]. However, in such a patient careful treatment should be done to prevent the progression of liver fibrosis. Finally, if the value of the slope is negative and also the last data of the mean FIB4 index YTD is less than 1.30 in the latest correlation, the risk of liver fibrosis is considered to be low.

We hope that the methods in this study will be the benefits to patients with NAFLD and in the future the methods will be compared to other markers and modalities for estimating liver fibrosis, with increased number of patients.

## 5. Conclusion

This study demonstrated that in patients with NAFLD the correlations between the period from the first to each examination date and the mean value of FIB4 index during the past one year to each examination date were strongly recognized. Approximately, the time-dependent change of FIB4 index and its increase-decrease rate per year could be speculated simply and accurately.

## Figures and Tables

**Table 1:** Characteristics of all 23 patients.

Patients (*n* = 23)	Laboratory findings		
	At the first examination	Peak value	At the last examination
		(bottom value only as for platelet count)	
Gender (male)		12 (52.2%)	
Age (years)	58.2 ± 8.5	NA	68.8 ± 9.5
AST (U/L)	40 ± 26	57 ± 30	28 ± 10
ALT (U/L)	49 ± 35	72 ± 35	26 ± 12
GGT (U/L)	NA	94 ± 83	45 ± 44
FIB4 index	1.66 ± 0.78	2.84 ± 1.34	2.04 ± 0.82
AAR	0.88 ± 0.22	1.52 ± 0.34	1.17 ± 0.31
Platelet count (×10^9^/L)	215 ± 68	165 ± 45	207 ± 63
Type IV collagen 7S (ng/mL)	NA	5.2 ± 2.0	4.4 ± 1.4
M2BPGi	NA	1.09 ± 0.86	0.88 ± 0.74

Mean ± SD			

Continuous variables were shown as mean ± standard deviation. At the last examination both type IV collagen 7S and M2BPGi were examined in all 23 patients. *n*, number of patients; NA, no analysis; AST, aspartate aminotransferase; ALT, alanine aminotransferase; GGT, gamma-glutamyl transpeptidase; AAR, AST/ALT ratio; M2BPGi, Mac-2 binding protein.

**Table 2:** All 23 main correlations.

Patients	Slope			*p*	*r*			*r* ^2^	Interval between examinations^#^		The main correlation's period	Ratio	Total analyzed period	Total clinical period
Case number	Positive value	Negative value	Absolute value		Positive value	Negative value	Absolute value		(years)	(days)	(years)	(%)	(years)	(years)
Case 1	0.4098		0.4098	2 × 10^−51^	0.98		0.98	0.96	0.12	44	8.2	64	12.8	13.9
Case 2	0.3343		0.3343	1 × 10^−20^	0.98		0.98	0.96	0.08	29	2.4	28	8.7	9.7
Case 3	0.2698		0.2698	5 × 10^−33^	0.96		0.96	0.92	0.18	66	9.5	94	10.1	11.1
Case 4	0.1923		0.1923	9 × 10^−8^	0.99		0.99	0.98	0.12	44	1.2	72	1.6	2.7
Case 5	0.1618		0.1618	0.01	0.96		0.96	0.92	0.19	70	1.1	32	3.3	4.4
Case 6	0.084		0.084	2 × 10^−8^	0.93		0.93	0.87	0.43	156	8.8	73	12.1	13.6
Case 7	0.0762		0.0762	9 × 10^−7^	0.82		0.82	0.67	0.19	71	3.4	35	9.6	10.9
Case 8	0.0758		0.0758	1 × 10^−42^	0.92		0.92	0.85	0.16	58	14.7	93	15.9	17.3
Case 9	0.0755		0.0755	3 × 10^−44^	0.91		0.91	0.83	0.09	35	9.9	63	15.8	17.1
Case 10	0.0708		0.0708	2 × 10^−53^	0.94		0.94	0.88	0.11	42	12	98	12.2	13.5
Case 11	0.0652		0.0652	4 × 10^−43^	0.96		0.96	0.91	0.15	54	9.4	84	11.2	12.5
Case 12	0.0629		0.0629	1 × 10^−32^	0.84		0.84	0.71	0.13	47	14.1	96	14.8	16
Case 13	0.0608		0.0608	6 × 10^−10^	0.8		0.8	0.64	0.27	97	9.1	80	11.4	12.5
Case 14	0.0349		0.0349	5 × 10^−8^	0.69		0.69	0.47	0.1	38	4.8	67	7.1	8.2
Case 15	0.0302		0.0302	1 × 10^−13^	0.74		0.74	0.55	0.2	72	13.9	98	14.2	15.3
Case 16	0.0299		0.0299	6 × 10^−11^	0.89		0.89	0.79	0.29	105	7.6	75	10.1	11.2
Case 17	0.0256		0.0256	6 × 10^−48^	0.95		0.95	0.9	0.11	40	9.5	61	15.5	16.6
Case 18		−0.089	0.089	0.004		−0.95	0.95	0.9	0.21	76	1.2	53	2.3	3.5
Case 19		−0.0984	0.0984	5 × 10^−12^		−0.91	0.91	0.82	0.11	39	2.9	43	6.8	7.9
Case 20		−0.1078	0.1078	0.005		−0.95	0.95	0.89	0.4	145	2	47	4.2	5.2
Case 21		−0.1237	0.1237	6 × 10^−5^		−0.94	0.94	0.88	0.13	46	1.3	63	2.1	3.1
Case 22		−0.307	0.307	2 × 10^−11^		−0.97	0.97	0.93	0.12	44	2	37	5.5	6.6
Case 23		−0.3681	0.3681	2 × 10^−11^		−0.95	0.95	0.9	0.12	45	3.2	27	11.9	12.9

Mean ± SD	0.1212 ± 0.1114	−0.1823 ± 0.1117	0.1371 ± 0.1147		0.90 ± 0.09	−0.94 ± 0.02	0.91 ± 0.08	0.83 ± 0.13	0.17 ± 0.09	64 ± 33	6.6 ± 4.5	64 ± 23	9.5 ± 4.5	10.7 ± 4.6

^#^Each value was shown in total clinical period; continuous variables were shown as mean ± standard deviation. Slope, the slope of the correlation; *p*, a *p* value; *r*, correlation coefficient; *r*^2^, a squared value of *r*; The main correlation's period, the period in which the main correlation was recognized; Ratio, the ratio of the main correlation's period to the total analyzed period; Total analyzed period, the period from the earliest examination date at least a year after the first examination to the last examination date; Total clinical period, the period from the first to the last examination date.

**Table 3:** All 41 correlations recognized in this study.

Patients	Phase with the before-main correlation							Phase with the main correlation		Phase with the after-main correlation						
Case number	Period (years)	Ratio (%)	Slope	*p*	*r*	Absolute value of *r*	*n*	Slope	*n*	Period (years)	Ratio (%)	Slope (%)	*p*	*r*	Absolute value of *r*	*n*
Case 1	1.1	8	−0.5133	0.0006	−0.91	0.91	9	0.4098	71	3.6	28	−0.2735	2 × 10^−17^	−0.95	0.95	33
Case 2	1.1	13	−0.4304	3 × 10^−9^	−0.96	0.96	16	0.3343	30	1.9	22	−0.2688	1 × 10^−14^	−0.95	0.95	28
Case 3	No existence							0.2698	59	No existence						
Case 4	No existence							0.1923	10	No existence						
Case 5	0.8	24	−0.2475	0.005	−0.97	0.97	5	0.1618	5	No recognition			(0.17)	(−0.83)	-	(4)
Case 6	Impossible to analyze						(2)	0.084	18	1.4	12	−0.3875	0.004	−0.98	0.98	5
Case 7	0.7	7	−0.082	0.0001	−0.99	0.99	5	0.0762	24	3	31	−0.0619	0.001	−0.85	0.85	11
Case 8	No recognition			(0.34)	(−0.86)	-	(3)	0.0758	101	No existence						
Case 9	No existence							0.0755	112	1.1	7	−0.3446	2 × 10^−7^	−0.94	0.94	15
Case 10	No existence							0.0708	113	No existence						
Case 11	Impossible to analyze						(2)	0.0652	80	No existence						
Case 12	No existence							0.0629	116	No existence						
Case 13	2	17	−0.1093	0.0008	−0.99	0.99	5	0.0608	40	No existence						
Case 14	1.4	20	−0.1118	1 × 10^−6^	−0.95	0.95	12	0.0349	49	No existence						
Case 15	No existence							0.0302	72	No existence						
Case 16	0.5	5	−0.1535	0.04	−0.998	0.998	3	0.0299	30	No recognition			(0.09)	(−0.82)	-	(5)
Case 17	3.4	22	−0.0401	1 × 10^−10^	−0.81	0.81	41	0.0256	96	No existence						
Case 18	No existence							−0.089	6	No recognition			(0.07)	(0.85)	-	(5)
Case 19	1	15	0.1512	1 × 10^−8^	0.98	0.98	13	−0.0984	30	0.7	11	0.1308	0.004	0.95	0.95	6
Case 20	No recognition			(0.27)	(0.73)	-	(4)	−0.1078	6	No existence						
Case 21	No existence							−0.1237	10	No existence						
Case 22	1.5	27	0.3595	6 × 10^−5^	0.9	0.9	12	−0.307	19	1.1	21	0.229	0.004	0.88	0.88	8
Case 23	No existence							−0.3681	22	4.8	41	0.1835	2 × 10^−14^	0.94	0.94	30

The mean ± SD	1.3 ± 0.7	16 ± 7				0.95 ± 0.05				1.9 ± 1.3	22 ± 11				0.93 ± 0.04	

Continuous variables were shown as mean ± standard deviation. Period, the period in which each correlation was recognized; Ratio, the ratio of each correlation's period to the total analyzed period; Slope, the slope of each correlation; *p*, a *p* value; *r*, correlation coefficient; *n*, number of data in each correlation; No existence, the correlation did not exist; Impossible to analyze, the analysis was impossible; No recognition, the correlation was not recognized statistically and both *p* and *r* were shown as figures in parentheses.

**Table 4:** All 14 new main correlations analyzed for the period newly set.

Case number of the patient	Slope	Absolute value of slope	*p*	Absolute value of *r*	*n*
Case 1	0.415	0.415	6 × 10^−10^	0.9	25
Case 2	0.4296	0.4296	9 × 10^−8^	0.97	13
Case 3	0.6245	0.6245	0.001	0.89	9
Case 6	0.1186	0.1186	0.045	0.82	6
Case 7	−0.1015	0.1015	0.035	0.9	5
Case 8	0.3461	0.3461	4 × 10^−11^	0.99	13
Case 9	0.4383	0.4383	1 × 10^−12^	0.99	16
Case 10	0.0921	0.0921	3 × 10^−9^	0.89	25
Case 11	0.1192	0.1192	1 × 10^−10^	0.96	19
Case 12	0.1062	0.1062	5 × 10^−16^	0.95	31
Case 14	−0.0458	0.0458	0.004	0.91	7
Case 15	−0.1094	0.1094	8 × 10^−6^	0.96	10
Case 17	0.1304	0.1304	0.004	0.88	8
Case 23	0.3707	0.3707	0.004	0.85	9

The mean ± SD		0.2462 ± 0.1766		0.92 ± 0.05	

Continuous variables were shown as mean ± standard deviation. In all the 23 patients the period was newly set from the closest date after half the total clinical period to that date after three-quarters. Of 23 patients, seven whose analyzed period remained less than two years were excluded. In the remaining 16 patients, the new main correlations were analyzed. In two of these 16, the correlations were not recognized. In case 16 the correlation could not be analyzed because of only two data and in case 13 number of data was three and the correlation was not recognized by *p* = 0.08 and *r* = 0.99. Slope, the slope of the correlation; *p*, a *p* value; *r*, correlation coefficient; *n*, number of data to analyze the correlation.
